# Information Geometry-Based Two-Stage Track-Before-Detect Algorithm for Multi-Target Detection in Sea Clutter

**DOI:** 10.3390/e27101017

**Published:** 2025-09-27

**Authors:** Jinguo Liu, Hao Wu, Zheng Yang, Xiaoqiang Hua, Yongqiang Cheng

**Affiliations:** College of Electronic Science and Technology, National University of Defense Technology, Changsha 410073, China; liujinguo19@nudt.edu.cn (J.L.);

**Keywords:** information geometry, radar target detection, multi-target detection, track-before-detect

## Abstract

To address the challenges of radar multi-target detection in marine environments, this paper proposes an information geometry (IG)-based, two-stage track-before-detect (TBD) framework. Specifically, multi-target measurements are first modeled on the manifold, leveraging its geometric properties for enhanced detection. The designed scoring function incorporates both the feature dissimilarity between targets and clutter, as well as the precise inter-target path associations. Consequently, a novel merit function combining feature dissimilarity and transition cost is derived to mitigate the mutual interference between adjacent targets. Subsequently, to overcome the integrated merit function expansion phenomenon, a two-stage integration strategy combining dynamic programming (DP) and greedy integration (GI) algorithms was adopted. To tackle the challenges of unknown target numbers and computationally infeasible multi-hypothesis testing, a target cancellation detection scheme is proposed. Furthermore, by exploiting the independence of multi-target motions, an efficient implementation method for the detector is developed. Experimental results demonstrate that the proposed algorithm inherits the superior clutter discrimination capability of IG detectors in sea clutter environments while effectively resolving track mismatches between neighboring targets. Finally, the effectiveness of the proposed method was validated using real-recorded sea clutter data, showing significant improvements over conventional approaches, and the signal-to-clutter ratio was improved by at least 2 dB.

## 1. Introduction

As one of the remote sensing observation tasks for marine surface environments, multi-target detection and tracking under low signal-to-clutter ratio (SCR) conditions presents significant challenges [1,2,3]. In multi-target detection and tracking scenarios, there are two main challenges [4]. First, weak targets are often submerged in strong clutter backgrounds [5,6,7]. Second, the spatial and motion coupling of adjacent targets can cause trajectory confusion [4,8]. In practical engineering applications, traditional multi-target tracking methods face significant performance bottlenecks in complex scenarios.

At the detection and tracking level, the classic approach is detect-before-track (DBT) [9,10]. By establishing detection thresholds, measurement points exceeding the threshold undergo subsequent tracking procedures, with representative methods including probabilistic data association (PDA) [11] and multiple hypothesis testing (MHT) [12]. However, such processing frameworks are suboptimal for target detection under low SCR conditions as the thresholding operation irreversibly discards weak target information. In contrast to conventional tracking methods, track-before-detect (TBD) technology embeds the tracking process within the detection framework, emerging as a core solution for weak target detection [13,14]. The superiority of TBD lies in its capacity for the joint processing of multi-frame echo measurements, leveraging spatiotemporal correlations of targets to achieve SCR improvements through energy integration. Dynamic programming (DP) [15,16], particle filtering (PF) [17], Hough transform [18], and the greedy integration (GI) algorithm [19] can all implement typical TBD algorithms. Extensive research has thoroughly demonstrated the effectiveness of TBD methods [20]. TBD technology can utilize not only target amplitude information, but also phase characteristics. For example, the logarithm of the complex measurement-based likelihood ratio (LCLR) incorporates complex-domain phase information and enhances extended target detection capabilities through intra-frame coherent integration [21]. Another major challenge in multi-frame joint processing in multi-target scenarios is the detection of adjacent targets [8,22]. Due to the mutual proximity of targets, the algorithm uncontrollably integrates the energy of adjacent targets during the integration of the merit function. This leads to targets becoming indistinguishable after multi-frame processing. Typical successive-target-cancellation Viterbi-like tracking algorithm (STC-VTA) [22,23,24] methods are based on the ideal assumption of sparse target spatial distribution, employing serial trajectory extraction or grouped processing detection strategies [25]. However, the DP-TBD algorithm suffers from the severe merit function expansion phenomenon, which leads to target energy diffusion and merging of peak envelopes from adjacent multiple targets. Therefore, traditional energy-based merit function designs exhibit significant performance degradation in detecting and tracking adjacent targets under strong clutter environments.

Due to the non-homogeneous nature of sea clutter and the limited availability of homogeneous sample data, the estimation accuracy of the clutter covariance matrix (CCM) is often compromised, consequently leading to degraded detection performance [26,27]. Currently, many studies have shown that information geometry (IG) methods applied to radar target detection problems can effectively address target detection issues in scenarios with limited pulses and non-homogeneous strong clutter [28,29,30]. For instance, in [31,32,33], the geometric properties of positive definite matrix space were fully exploited to achieve robust estimation of disturbance covariance matrices through geometric barycenters or geometric medians. The results demonstrate that such geometric approaches yield significant performance improvements in non-homogeneous clutter environments [34,35]. The core idea of information geometry methods is to model radar signals as geometric structures on Hermitian positive definite (HPD) manifold [36] or power spectrum (PS) manifold [37]. Early studies were based on the Riemannian distance, but this was computationally complex. Subsequent developments introduced various efficient geometric metrics such as the Kullback–Leibler (KL) divergence and Bregman divergence, and adaptive mechanisms were then incorporated to optimize the detection performance in different clutter environments [38]. Currently, IG methods demonstrate unique advantages in detecting nonhomogeneous clutter, Doppler-overlapping targets, and weakly fluctuating targets, and they have been successfully applied in practical scenarios, such as ship detection and wake monitoring [39,40]. Currently, research on IG detectors has been extended to multi-frame joint processing. Existing IG-based TBD methods primarily focus on single-target detection [41,42,43]. However, in practical applications, there are usually multiple targets, and the merit function expansion phenomenon is exacerbated in multi-target scenarios. Therefore, it is necessary to research the IG-based TBD algorithm for multi-target scenarios.

This paper provides a new feasible solution for detecting and tracking multi-targets in sea clutter environments. Compared with previous single-target research [43], this paper addresses the core challenges in multi-target environments, particularly the issue of distinguishing adjacent targets, by redesigning the value function architecture based on information geometry. On one hand, we combine the DP algorithm with the GI algorithm to integrate the discriminative features between targets and clutter on the manifold, thereby suppressing the merit function expansion phenomenon. On the other hand, by leveraging the precise trajectory assignment capability of IG methods for different targets, a target cancellation strategy is adopted to achieve accurate tracking of adjacent multiple targets. These improvements establish the proposed method as a significant advancement, effectively addressing the more practical and challenging problem of multi-target detection and tracking. The main contributions of this paper are summarized as follows.

- A multi-target merit function integrating feature dissimilarity and transition cost is designed for multi-target scenarios, particularly for adjacent targets. On the manifold, the feature dissimilarity between targets and clutter is maximized, while the feature dissimilarity of the same target across adjacent frames is minimized. This ensures accurate matching of true multi-target trajectories and prevents track confusion in multi-frame scenarios with adjacent targets. Subsequently, a target cancellation method is employed to extract trajectories sequentially, achieving precise tracking of multiple targets in strong clutter backgrounds.
- Since the DP algorithm inherently leads to merit function expansion, and as adjacent targets further exacerbate energy diffusion, this paper employed a GI algorithm to suppress merit function expansion. First, the DP algorithm integrates merit function over a certain number of frames, allowing true target states to integrate higher values. Then, the GI algorithm is applied, where states with the highest merit functions are prioritized for processing while suppressing their use by other integration paths. This two-stage integration method effectively prevents redundant usage of target information, thereby mitigating merit function expansion.
- The multi-target detection and tracking performance of the proposed algorithm was verified using real sea clutter data. For both adjacent parallel and crossing targets, the proposed algorithm demonstrated superior detection performance, with a SCR improvement exceeding 2 dB. In complex multi-target scenarios, the proposed algorithm also achieved higher accuracy in target trajectory estimation.

The remainder of this paper is organized as follows. Section 2 presents the background and problem formulation. The preliminaries of information geometry detectors are developed in Section 3. Section 4 focuses on the design of multi-target merit functions. The multi-target, multi-frame processing framework is detailed in Section 5. Experimental validation and discussion are presented in Section 6, followed by concluding remarks in Section 7.

## 2. Background and Problem Formulation

This section mainly introduces the measurement model and target model, as well as proposes a multi-target, multi-frame detection problem.

### 2.1. Measurement Model and Target Model

**Figure 1:**
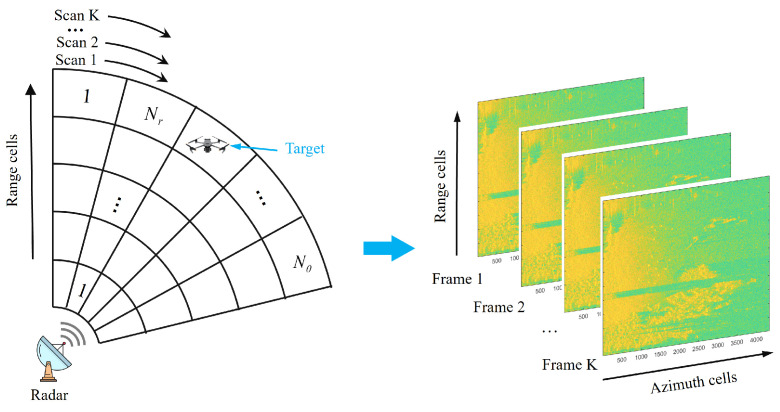
Multi-frame range and azimuth measurements in radar systems.

Assume that a radar system uses a scanning mode to monitor low-altitude targets. Specifically, during the observation period, the radar performs *K* scans of the observation area and collects *K* frames of echo data. The echo data are two-dimensional measurements in the range-azimuth domain, as shown in Figure 1. The observation plane is divided into $N_{r}\times N_{a}$ resolution cells, meaning each frame contains $N_{r}$ range cells and $N_{a}$ azimuth cells. For each frame of data, $Z_{k}=\left[z_{k}^{1,1},\ldots ,z_{k}^{r,a},\ldots ,z_{k}^{N_{r},N_{a}}\right]\in R_{+}^{N_{r}\times N_{a}}$ with $k=1,\ldots ,K,\;r=1,\ldots ,N_{r}$ and $a=1,\ldots ,N_{a}$. Each azimuth cell contains *n* pulses, which is a coherent processing interval (CPI), where $\Delta_{r}$ is the minimum range resolution and $\Delta_{a}$ is the minimum azimuth resolution. For *K* consecutive scan frames, the data is represented as $Z_{1:K}=\left[Z_{1},\ldots ,Z_{K}\right]$.

In a multi-target scenario, assume that there are *M* targets simultaneously present within the observation area. Consider the *m*-th (m=1,…,M) target moving independently in a two-dimensional plane. Each target can be modeled independently in the state space $R^{s}$. The *m*-th target at the *k*-th frame can be represented as $x_{k}^{m}={\left[r_{k}^{m},a_{k}^{m}\right]}^{T}\in R^{2}$, where $r_{k}^{m}$ and $a_{k}^{m}$ represent the position of the target in the range-azimuth domain. It should be noted that the dimension s=2 here, but the dimension *s* can be directly generalized. The state of each target at each frame can be cascaded from the states of all targets at that frame. Specifically, the state of each target at the *k*-th frame is $X_{k}^{1:M}={\left[x_{k}^{1},x_{k}^{2},\ldots ,x_{k}^{M}\right]}^{T}$, and the state of each target at the *K* frames is $X_{1:K}^{1:M}=\left[X_{1}^{1:M},X_{k}^{1:M},\ldots ,X_{K}^{1:M}\right]$.

We assume that the target size occupies only one resolution cell. For each range cell, the echo data in a CPI is represented as $z={\left[z_{1},z_{2},\ldots ,z_{n}\right]}^{T}\in C^{n}$, where z is an n-dimensional complex vector. In a CPI, the echo data containing the target can be expressed as

(1) z=αp+c,

where p represents the target steering vector; α represents the target amplitude; and $p={\left[1,e^{-j2\pi f_{d}},\ldots ,e^{-j2\pi f_{d}\left(n-1\right)}\right]}^{T}$ and $f_{d}$ represent the normalized Doppler frequency.

Assuming that the inter-frame state transition of the target follows a first-order Markov model and the process noise is Gaussian-distributed, the state transition equation of the target is

(2) $$x_{k}^{m}|x_{k-1}^{m}∼N\left(\cdot ;Fx_{k-1}^{m},Q\right),$$

where N(x;μ,Σ) represents a Gaussian distribution, with mean μ and covariance matrix Σ, while the target state transition matrix F and process noise variance matrix Q are represented as follows:

(3) $$F=\left[\begin{array}{cc} 1 & T\\ 0 & 1 \end{array}\right]⊗I_{2},$$

(4) $$Q=\kappa \left[\begin{array}{cc} T^{3}/3 & T^{2}/2\\ T^{2}/2 & T \end{array}\right]⊗I_{2}.$$

Here, *T* represents the time interval between two frames; $I_{2}$ denotes a 2×2 identity matrix; and ⊗ is used to represent the direct product of matrices. The parameter κ is used to measure the intensity of process noise in the system. The algorithm proposed in this paper is not limited to the model in (2). For targets with complex motion characteristics, more accurate simulation can be achieved by introducing more advanced motion models [44,45]. The proposed method remains applicable.

In radar systems, due to resolution limitations, we cannot precisely determine the actual continuous state of a target [46]. Therefore, we typically discretize the target’s state. For example, in a two-dimensional space, if we divide the space into a grid, each grid point can be considered a “resolution cell”. This method is a simplification that maps the continuous target state onto discrete cells, thereby enabling us to more easily process and analyze the target’s state. The above measurement model and target model are based on this.

### 2.2. Problem Formulation

Based on the above model assumptions, when there are *M* targets in the observation area, the detection model for the *k*-th frame of received data $z_{k}^{r,a}$ (where $r=1,\ldots ,N_{r}$ denotes the range cell, $a=1,\ldots ,N_{a}$ represents the azimuth cell and k=1,…,K indicates the frame index) can be expressed as

(5) $$\left\{\begin{array}{c} H_{0}:z_{k}^{r,a}=w_{k}^{r,a},\\ H_{1}:z_{k}^{r,a}=\left\{\begin{array}{cc} \alpha_{1}p_{1}+w_{k}^{r_{1},a_{1}}, & \mathrm{Target}\;1\;\mathrm{in}\;\mathrm{cell}\;(r_{1},a_{1}),\\ \vdots \\ \alpha_{M}p_{M}+w_{k}^{r_{M},a_{M}}, & \mathrm{Target}\;M\;\mathrm{in}\;\mathrm{cell}\;(r_{M},a_{M}),\\ w_{k}^{r,a}, & \mathrm{otherwise}. \end{array}\right. \end{array}\right.$$

where $H_{0}$ and $H_{1}$ represent the absence and presence of targets in the $\left(r_{m},a_{m}\right)$ resolution cell, respectively; $w_{k}$ represents clutter data; and $\alpha_{m}p_{m}$ represents target data.

In scenarios where the number of targets is unknown, the purpose of the algorithm proposed in this paper is to estimate the number of targets in the observation area and their states. The determination of the target existence and subsequent estimation of target quantity are achieved by thresholding the detection statistic.

Based on the above multi target, multi-frame detection model, the following content will propose corresponding multi-frame processing strategies.

## 3. Preliminaries of Information Geometry Detector

For a complex signal vector $z={\left[z_{1},z_{2},\ldots ,z_{n}\right]}^{T}$ in a CPI containing *n* pulses, its theoretical covariance matrix is defined as

(6) $$C=E[zz^{H}],$$

where E[·] denotes expectation or statistical average, and $z^{H}$ is the Hermitian transpose of z. After unfolding, the matrix element ${\left[C\right]}_{i,j}$ represents the correlation coefficient between the *i*-th and *j*-th pulses:

(7) $${\left[C\right]}_{i,j}=E\left[z_{i}{\bar{z}}_{j}\right],$$

where ${\bar{z}}_{j}$ is the complex conjugate of $z_{j}$.

The theoretical covariance matrix reflects the statistical characteristics of the signal but requires an infinite number of samples. Since only a finite number of samples can be obtained in practice, the sample covariance matrix must be used to estimate C. Assuming that there are *N* independent and identically distributed reference cell data ${\left\{z_{i}\right\}}_{i=1}^{N}$, the estimation method is as follows:

(8) $$C=\frac{1}{N}\sum_{i=1}^{N}z_{i}z_{i}^{H}.$$

For stationary signals, the covariance matrix has Toeplitz properties, which can simplify calculations using correlation coefficients. For a single observation z, calculate the correlation coefficient with lag *l*:

(9) $${\hat{c}}_{i}=\frac{1}{n}\sum_{j=0}^{n-i-1}z_{j}{\bar{z}}_{j+i},0\leq i\leq n-1.$$

Constructing Toeplitz matrices, we have the following:

(10) $$C=E\left[zz^{H}\right]=\left[\begin{array}{ccccccc} {\hat{c}}_{0} & \; & {\bar{\hat{c}}}_{1} & \; & \ldots & \; & {\bar{\hat{c}}}_{n-1}\\ {\hat{c}}_{1} & \; & {\hat{c}}_{0} & \; & \ldots & \; & {\bar{\hat{c}}}_{n-2}\\ \vdots & \; & \ddots & \; & \ddots & \; & \vdots \\ {\hat{c}}_{n-1} & \; & {\hat{c}}_{n-2} & \; & \ldots & \; & {\hat{c}}_{0} \end{array}\right].$$

Here, C is the Toeplitz HPD matrix that satisfies $C=C^{H}$.

The HPD manifold $P^{+}\left(n\right)$ is a nonlinear space composed of all n×n Hermitian positive definite matrices, with a Riemannian geometric structure, as follows:

(11) $$P^{+}\left(n\right)=\left\{C\in P\left(n\right)|C=C^{H},C≻0\right\},$$

where P(n) denotes the set of n×n Hermitian matrices. When C≻0, it means that $\forall z\in C^{n}\setminus \left\{0\right\}$, $z^{H}Cz>0$.

For the matrix information geometry (MIG) detector, the received signals are modeled as HPD matrices. These matrices can be regarded as points on the HPD manifold. The IG detector transforms the target detection problem into a classification problem on the HPD manifold and utilizes its inherent geometric properties to derive detection strategies [29,36]. Consequently, the decision derived from the geometric dissimilarity between the cell under test (CUT) covariance matrix $C_{\mathrm{CUT}}$ and the geometric mean $\bar{C}$ of the reference cells can be expressed as

(12) $$\phi_{D}\left(C_{\mathrm{CUT}},\bar{C}\right)\underset{H_{0}}{\overset{H_{1}}{≷}}\eta ,$$

where $\phi_{D}$ represents the geometric distance between two points on the HPD manifold, and η is the detection threshold determined at a fixed false alarm rate. By calculating the geometric mean $\bar{C}$ of the covariance matrices of reference cells $C_{1},C_{2},\ldots ,C_{N_{R}}$ as a statistical representation of the background clutter, we have

(13) $$\begin{array}{cc} \bar{C} & =G(C_{1},C_{2},\ldots ,C_{N_{R}})\\ \; & =\arg\min_{C\in P^{+}\left(n\right)}\frac{1}{N_{R}}\sum_{i=1}^{N_{R}}\phi_{D}\left(C,C_{i}\right), \end{array}$$

where $N_{R}$ denotes the number of reference cells.

**Figure 2:**
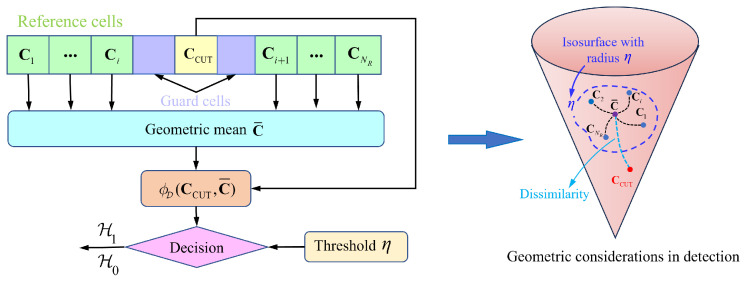
The framework of the MIG detector.

Figure 2 shows the working framework of the MIG detector. The MIG detector determines whether the target exists by judging whether the $C_{\mathrm{CUT}}$ is located inside the isosurface with center $\bar{C}$ and radius η. This isosurface is closely related to the ability to distinguish between the target and noise.

To calculate the geometric distance between two points on a manifold, the Riemannian distance is a geodesic metric on HPD manifolds. However, due to the computational complexity of the Riemannian distance and the need for iteration to calculate the mean, the computational cost of the Riemannian metric is high. An alternative is information divergence, with the most commonly used being the Kullback–Leibler (KL) divergence [38]. The mean of the KL distance has an analytical expression and is simple to compute. Research has demonstrated that the KL divergence performs well as a distance metric on manifolds [37], which is defined as

(14) $$\phi_{D_{\mathrm{KL}}}\left(C_{1},C_{2}\right)=\mathrm{tr}\left(C_{2}^{-1}C_{1}-I\right)-\log\det\left(C_{2}^{-1}C_{1}\right),$$

and the geometric mean $\bar{C}$ is

(15) $$\begin{array}{cc} \bar{C} & =G(C_{1},C_{2},\ldots ,C_{N_{R}})\\ \; & ={\left(\frac{1}{N_{R}}\sum_{i=1}^{N_{R}}C_{i}^{-1}\right)}^{-1}. \end{array}$$

As another IG detector with excellent detection performance, the power spectrum information geometry (PSIG) detector is based on the duality between the Toeplitz HPD manifold and the PS manifold, and it is derived using the MIG detector induced by affine-invariant geometric measurements [37,42]. The PSIG detector has a broader application prospect due to its simpler computation and lower feature dimension. Here, the specific derivation process will not be presented. Similarly, utilizing the geometric structure of the PS manifold, the derived decision can be expressed as

(16) $$\psi_{D}\left(P_{\mathrm{CUT}},\bar{P}\right)\underset{H_{0}}{\overset{H_{1}}{≷}}\eta^{\prime },$$

where $\psi_{D}$ represents the geometric distance between two points on the PS manifold, and $\eta^{\prime }$ is the detection threshold determined at a fixed false alarm rate.

## 4. IG-Based Multi-Target Merit Function

**Figure 3:**
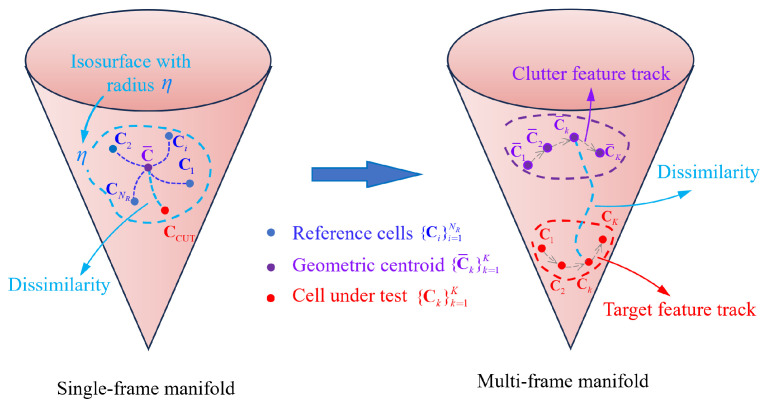
Schematic diagram of single-frame manifolds and multi-frame manifolds.

Based on the principles of information geometry detection, after modeling the data onto a manifold, it is necessary to distinguish the targets from the clutter based on geometric features. During single-frame processing, if the targets and clutter exhibit clear distinguishability on the manifold, the targets are located outside the isosurface with the clutter mean as its geometric center, as shown in Figure 2. Then, the targets can be easily detected. However, in strong clutter scenarios, the targets are typically close to the clutter center and located inside the isosurface. This results in poor geometric distinguishability, and single-frame information cannot detect the target, as shown in Figure 3 (Left). Therefore, it is necessary to consider the integration of multi-frame information. By integrating the differences between the targets and clutter across multiple frames, the dissimilarity between target tracks and clutter tracks increases, as shown in Figure 3 (Right). Thus, targets and clutter can be distinguished, improving detection performance.

For the sake of convenience in subsequent derivations, the following content will be based on TBD using MIG, as PSIG-based TBD and MIG-based TBD are similar. The subsequent content will use C and $\phi_{D}$ instead of P and $\psi_{D}$ for derivation.

**Figure 4:**
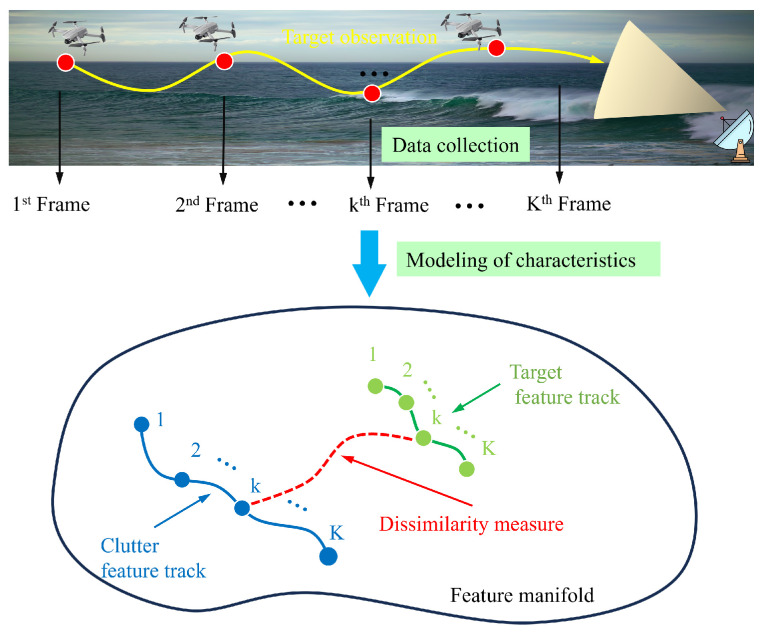
Single target multi-frame feature variations on the feature manifold.

The purpose of multi-frame TBD is to detect targets and output target trajectories simultaneously. The input of the IG-based TBD algorithm is the covariance matrix or power spectrum of the echo data. By analyzing the statistical characteristics of the targets and clutter, the clutter feature tracks and target feature tracks can be constructed on the manifold, as shown in Figure 4. The dissimilarity between the clutter zone and the target zone is integrated as a detection statistic.

Taking the *m*-th target as an example, assume that its trajectory is $X_{1:K}^{m}=\left[x_{1}^{m},x_{k}^{m},\ldots ,x_{K}^{m}\right]$. After the *k*-th frame of echo data is modeled using manifold modeling, the CUT is modeled as $C_{\mathrm{CUT}}^{m}\left(r_{k},a_{k}\right)$, and the geometric center of the reference cells is modeled as ${\bar{C}}^{m}\left(r_{k},a_{k}\right)$, which is in the *r*-th range cell and *a*-th azimuth cell. Mapping all *K* frames of echo data onto the manifold will form two feature tracks on the manifold, as shown in Figure 4. The *K* frames of the CUT will form the target feature track $C_{\mathrm{CUT}}^{m}\left(r_{k},a_{k}\right),k=1,\ldots ,K$, while the geometric center of the reference cells will form the clutter feature track ${\bar{C}}^{m}\left(r_{k},a_{k}\right),k=1,\ldots ,K$. Therefore, the purpose of multi-frame integration is to maximize the dissimilarity between the target and clutter feature tracks.

Firstly, the range of motion between frames determines the interconnection of target states. We need to define the range of target state transitions between frames based on the target’s motion characteristics. In this paper, we used the target’s maximum velocity to constrain its state. Assume that the maximum velocity of the target within the observation area does not exceed $v_{max}$. Therefore, the set of target states of k−1-th frame that can be transferred to the target state $x_{k}^{m}$ can be represented as

(17) $$\tau \left(x_{k}^{m}\right)=\left\{{\left[\begin{array}{c} r_{k-1},a_{k-1} \end{array}\right]}^{T}\in R^{2}:\begin{array}{cc} |r_{k}-r_{k-1}| & \leq \tau_{1}\Delta_{r},\\ |a_{k}-a_{k-1}| & \leq \tau_{2}\Delta_{a} \end{array}\right\},$$

where $\tau_{1}=\left\lceil Tv_{max}/\Delta_{r}\right\rceil$, $\tau_{2}=\left\lceil Tv_{max}/\Delta_{a}\right\rceil$, $r_{k}$ and $a_{k}$ represent the range cell and azimuth cell positions in the *k*-th frame, respectively. Evidently, if $v_{max}$ is chosen appropriately, the resulting set of candidate trajectories can cover all possible maneuver paths. In cases involving more complex target maneuvers, the possible state transition set can be extended to higher dimensions. The algorithm proposed in this paper is also applicable.

In the case of multiple targets, the alternative hypotheses are $H_{0}$ and $H_{1}$, and the detection statistic requires estimation of the state sequence of multiple targets. A feasible method is to discretize the target state space and then enumerate possible multiple target paths to maximize the dissimilarity between the target and the clutter. Based on this principle, the target trajectories for 2≤k≤K can be estimated by the following equation:

(18) $${\hat{X}}_{2:K}^{1:M}=\arg\max_{\begin{array}{c} X_{k-1}^{1:M}\in \tau \left(X_{k}^{1:M}\right) \end{array}}\sum_{k=2}^{K}\phi_{D}\left(C_{\mathrm{CUT}}^{1:M}\left(r_{k},a_{k}\right),{\bar{C}}^{1:M}\left(r_{k},a_{k}\right)\right).$$

From (18), it can be seen that the statistic $\phi_{D}\left(C_{\mathrm{CUT}}^{1:M}\left(r_{k},a_{k}\right),{\bar{C}}^{1:M}\left(r_{k},a_{k}\right)\right)$ describes the distinguishing characteristics between multiple targets and clutter within a frame. However, due to the lack of specific state assignment relationships between multiple paths $X_{k-1}^{1:M}$, and between the set $X_{k-1}^{1:M}$ and the set $X_{k}^{1:M}$, the estimation of parameters ${\hat{X}}_{2:K}^{1:M}$ in (18) may encounter problems of incorrect association of multiple target trajectories.

To address the issue of tracking interference between multiple targets in adjacent frames, it is necessary to describe the precise allocation relationship between $X_{k-1}^{1:M}$ and $X_{k}^{1:M}$ when calculating multi-frame detection statistics. The interframe correlation path for the same target needs to be considered. On the manifold, CUTs of the same target in different frames are similar, and these points should be as close as possible. Due to the independence of multi-target motion, we obtained the following:

(19) $$\arg\min_{\begin{array}{c} X_{k-1}^{1:M}\in \tau \left(X_{k}^{1:M}\right) \end{array}}\sum_{k=2}^{K}\phi_{D}\left(C_{\mathrm{CUT}}^{1:M}\left(r_{k},a_{k}\right),C_{\mathrm{CUT}}^{1:M}\left(r_{k-1},a_{k-1}\right)\right).$$

This is a transition cost function that can suppress the trajectory exchange and trajectory confusion between multiple frames in a multi-target environment. This is because the inter-frame target correlation term imposes a large penalty on incorrect trajectory correlation.

The target motion is a first-order Markov process, so the target state transition is only related to the adjacent target states. By introducing the transition cost function (19) into (18) without changing the overall form of the trajectory estimation equation, we can obtain

(20) $${\hat{X}}_{2:K}^{1:M}=\arg\max_{\begin{array}{c} X_{k-1}^{1:M}\in \tau \left(X_{k}^{1:M}\right) \end{array}}\left(\sum_{k=2}^{K}\phi_{D}\left(C_{\mathrm{CUT}}^{1:M}\left(r_{k},a_{k}\right),{\bar{C}}^{1:M}\left(r_{k},a_{k}\right)\right)-\xi \sum_{k=2}^{K}\phi_{D}\left(C_{\mathrm{CUT}}^{1:M}\left(r_{k},a_{k}\right),C_{\mathrm{CUT}}^{1:M}\left(r_{k-1},a_{k-1}\right)\right)\right),$$

where ξ is the weighting factor. In (20), the inter-frame target correlation term is integrated into the multi-frame detection statistic to address the correlation issue of multi-target trajectories. When it matches the true multi-target path, it will take the maximum value. Now, the detection statistic not only includes intra-frame features for distinguishing from clutter, but also includes association weights for different path hypotheses. Therefore, the estimated value ${\hat{X}}_{2:K}^{1:M}$ under hypothesis $H_{1}$ is the multi-target tracking result with the largest association weight.

Therefore, we can obtain an IG-based scoring function

(21) $$\begin{array}{c} S\left(X_{k}^{1:M}\right)=\left\{\begin{array}{cc} \phi_{D}\left(C_{\mathrm{CUT}}^{1:M}\left(r_{k},a_{k}\right),{\bar{C}}^{1:M}\left(r_{k},a_{k}\right)\right)-\xi \phi_{D}\left(C_{\mathrm{CUT}}^{1:M}\left(r_{k},a_{k}\right),C_{\mathrm{CUT}}^{1:M}\left(r_{k-1},a_{k-1}\right)\right), & k>1,\\ \phi_{D}\left(C_{\mathrm{CUT}}^{1:M}\left(r_{k},a_{k}\right),{\bar{C}}^{1:M}\left(r_{k},a_{k}\right)\right), & k=1. \end{array}\right. \end{array}$$

By jointly processing multiple frames of data, an IG-based merit function can be obtained

(22) $$M\left(X_{2:k}^{1:M}\right)=\max_{\begin{array}{c} X_{k-1}^{1:M}\in \tau \left(X_{k}^{1:M}\right) \end{array}}\sum_{i=2}^{k}S\left(X_{k}^{1:M}\right),$$

where $M\left(X_{1}^{1:M}\right)=S\left(X_{1}^{1:M}\right)$.

After obtaining the merit function, classical single-target processing methods typically utilize dynamic programming (DP) techniques to obtain the optimal target trajectory. In the next section, we will use a more efficient path search method for detection.

## 5. Multi-Target Multi-Frame Processing Framework Design

This section uses the DP algorithm and greedy integration algorithm to integrate merit functions, and it then designs a multi target, multi-frame detector based on the target cancellation algorithm. The following is a detailed introduction to the design and processing flow of the detector.

### 5.1. Two Stage Integration of the Multiframe Maximization

For the TBD implementation process under strong clutter conditions, the global optimality characteristic of DP search can lead to severe expansion of the merit function [19]. The greedy integration (GI) algorithm has been proven to effectively alleviate the merit function expansion problem. This will facilitate the output of more accurate target trajectories and better detection performance [47]. Therefore, this paper will combine the DP and GI algorithm to implement target path search.

The idea works by first employing the DP algorithm to perform a globally optimal search over the first λ frames. The purpose of this step is to enhance the detectability of targets submerged in strong clutter by leveraging inter-frame information integration, thereby increasing the discriminability between targets and clutter. Subsequently, a greedy integration algorithm is applied to perform a locally optimal search on the integrated merit function obtained from the DP processing. This step simultaneously suppresses the integration of clutter-induced branching paths, effectively reducing the interference from clutter. This combined strategy ensures robust target detection while minimizing the false alarms caused by clutter.

The subsequent content will elaborate on the implementation details of merit function integration and path search.

(1) DP integration: For the first λ frames of echo measurements, after data preprocessing, the echoes are modeled as either covariance matrices or power spectra. Subsequently, we define the integrated merit function for $X_{k}^{1:M}$ at 2≤k≤λ frame as follows:

(23) $$M\left(X_{k}^{1:M}\right)=\max_{\begin{array}{c} X_{k-1}^{1:M}\in \tau \left(X_{k}^{1:M}\right) \end{array}}M\left(X_{k-1}^{1:M}\right)+S\left(X_{k}^{1:M}\right),$$

where $M\left(X_{1}^{1:M}\right)=S\left(X_{1}^{1:M}\right)$ at k=1 frame. Meanwhile, the backtracking function for target trajectories at 1≤k≤λ−1 frame can be defined as

(24) $${\hat{X}}_{k}^{1:M}=\arg\max_{\begin{array}{c} X_{k}^{1:M}\in \tau \left(X_{k+1}^{1:M}\right) \end{array}}M\left(X_{k}^{1:M}\right),$$

where ${\hat{X}}_{\lambda }^{1:M}=\arg\max_{\begin{array}{c} X_{\lambda }^{1:M}\in R^{s} \end{array}}M\left(X_{\lambda }^{1:M}\right)$ at λ frame.

(2) Greedy integration: The greedy integration algorithm is governed by two key operations—(1) preferential processing of maximal integration values, and (2) active suppression of clutter-induced branching paths.

For the λ+1≤k≤K frame, the integrated merit function is defined as follows.

Step 1: Find the state that reaches the maximum value of the integrated merit function in the k−1-th frame integrated merit function:

(25) $${\tilde{x}}_{k-1}^{1:M}=\arg\max_{\begin{array}{c} X_{k-1}^{1:M}\in R^{s} \end{array}}M\left(X_{k-1}^{1:M}\right).$$

The purpose of this operation is to prioritize states with larger integrated merit function values.

Step 2: Find the maximum value of the scoring function within the state transition range of the *k*-th frame corresponding to the ${\tilde{x}}_{k-1}^{1:M}$:

(26) $${\overset{ˇ}{x}}_{k}^{1:M}=\arg\max_{\begin{array}{c} X_{k}^{1:M}\in \tau^{-1}\left({\tilde{x}}_{k-1}^{1:M}\right) \end{array}}S\left(X_{k}^{1:M}\right).$$

The purpose of this operation is to prioritize states with higher scoring function values in frame *k*.

Step 3: Calculate to obtain the new integrated merit function as follows:

(27) $$M\left({\overset{ˇ}{x}}_{k}^{1:M}\right)=M\left({\tilde{x}}_{k-1}^{1:M}\right)+S\left({\overset{ˇ}{x}}_{k}^{1:M}\right).$$

Step 4: Clear the corresponding integrated merit function value and measurements:

(28) $$M({\tilde{x}}_{k-1}^{1:M})=0,$$

(29) $$Z_{k}\left({\overset{ˇ}{x}}_{k}^{1:M}\right)=0.$$

This step indicates that the integrated merit function for each state in frame *k* does not come from the same state in frame k−1. Therefore, the true target state does not spread to other states, thereby suppressing the expansion of the integrated merit function.

Step 5: Repeat Step 1 to Step 4 until all integrated merit functions $M(X_{k-1}^{1:M})$ at k−1 frame have been processed.

Based on the above process, the calculation of the multi-frame integrated merit function can be achieved. The following content will introduce how to implement multi-target detection and trajectory extraction.

### 5.2. Multi-Target Multi-Frame Detector Design

TBD in a multi-target state involves high-dimensional maximization solutions. However, in multi-target detection scenarios, when the number of targets *M* is unclear or large, directly applying the multi-target DP-TBD algorithm for trajectory estimation, as shown in (20), requires enumerating all possible values of *M* and their corresponding trajectories, which is computationally infeasible [22,25]. Due to the uncertainty of *M*, the system must perform joint hypothesis testing on all possible *M* values, which significantly increases the computational load and implementation difficulty of the algorithm. Even when using multi-path dynamic programming algorithms and their improved methods (such as STC-VTA and single-pass STC-VTA) [23], their computational complexity still increases exponentially with the upper limit of the number of targets $M_{max}$ [8].

To address the aforementioned estimation problem in (20), we propose a target cancellation path search algorithm. The core concept of this approach is to initially perform integrated merit function under a single-target hypothesis $H_{1}$ when no prior target information is available [4]. After each integration step, only the optimal target state is searched and its trajectory is estimated and stored. Subsequently, the echo measurement information associated with this target is removed, and the process of optimal target state search is repeated iteratively. This procedure continues until all targets are completely detected. Therefore, the multi-target TBD problem for m=1,…,M can be re-expressed as follows:

(30) $${\hat{X}}_{K}^{m}=\arg\max_{\begin{array}{c} X_{K}^{m}\in R^{s} \end{array}}M\left(X_{K}^{m}\right),$$

(31) $$s.t.M\left({\hat{X}}_{K}^{m}\right)>\eta_{K}.$$

**Figure 5:**
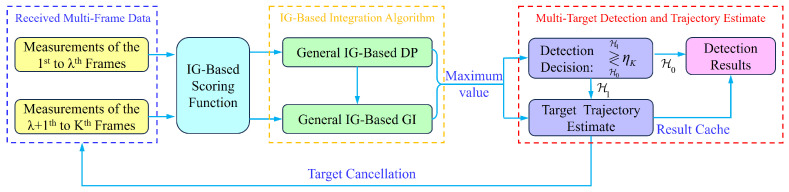
The general framework of an IG-based multi-target TBD.

The threshold $\eta_{K}$ is determined through Monte Carlo simulations using non-target data under a constant false alarm rate. The target number is estimated based on the number of times the integrated merit function exceeds the threshold $\eta_{K}$. The target trajectory is derived from the equations provided in Section 5.1. The complete multi-target detection process is illustrated in Figure 5.

To enable a fast implementation of the algorithm, the target region can be clustered. The target path is divided into several isolated, indivisible clusters, thereby decomposing the complex multi-target joint optimization problem into multiple independent subproblems. First, the target cluster structure is identified through clustering processing, and then a multi-frame detector is used to detect and track each cluster target in parallel.

Under hypothesis $H_{1}$, the merit function integration is performed on the *K* frame echo measurements $Z_{1:K}$. Then, centered on the portions of the integrated merit function plane that exceed the threshold $\eta_{K}$, the observation region is partitioned into *j* sub-regions:

(32) $$J=\left\{X_{1:K}^{1}:M\left(X_{1:K}^{1}\right)>\eta_{K}\right\}.$$

The estimated trajectories $\hat{T}$ obtained through the set J can be formally expressed as follows:

(33) $$\hat{T}=T_{t}\cup T_{c},$$

where $T_{t}$ denotes true target trajectories, and $T_{c}$ represents clutter-induced trajectories. Due to the integrated merit function expansion phenomenon, a subset of trajectories $T_{l}\subseteq \hat{T}$ exhibit shared root paths $\rho_{l}$, satisfying the following:

(34) $$\rho_{l}=\underset{\chi \in T_{l}}{⋂}\chi \;\;\mathrm{with}\;\;M\left(\rho_{l}\right)>M\left(\chi \right),\forall \chi \in T_{l}\setminus \rho_{l},$$

where $\chi \in T_{l}\setminus \rho_{l}$ are called branching trajectories, and the root path $\rho_{l}$ has a larger integrated merit function.

The observation area Ω is partitioned into non-overlapping subregions ${\left\{\Omega_{l}\right\}}_{l=1}^{L}$ according to root path locations:

(35) $$\Omega =\overset{L}{\underset{l=1}{⋃}}\Omega_{l}\;s.t.\;\Omega_{p}\cap \Omega_{q}=\emptyset \left(p\neq q\right).$$

Within each $\Omega_{l}$, the multi-frame TBD process operates independently, with each subregion containing $m_{l}\geq 1$ targets and $\sum_{l=1}^{L}m_{l}=M$.

Therefore, the maximum, integrated merit function of multi-target state estimation can be expressed as the sum of the multi-target state estimation of each subregion:

(36) $$\max_{X_{k}\in R^{s}}M\left(X_{1:K}^{1:M}\right)=\sum_{l=1}^{L}\max_{X_{k}\in R^{s}\left(\Omega_{l}\right)}M\left(X_{1:K}^{1:m_{l}}\right).$$

This strategy effectively reduces the complexity of multi-target detection while maintaining processing efficiency through path clustering and problem decomposition. Through recursive solving in Algorithm 1, targets can be extracted one by one from the subspace of the state space.
**Algorithm 1:** IG-based multi-target TBD algorithm with target cancellation
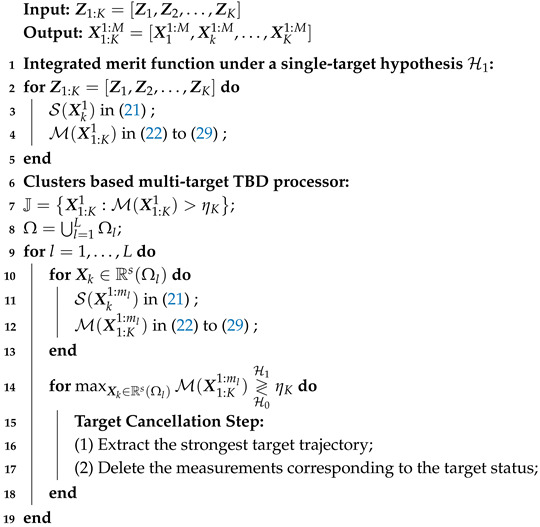


### 5.3. Computational Complexity

In the process of computing the IG-based detection statistic, the computational complexity primarily stems from the scoring function and the merit function. The computational complexity of the MIG detector and PSIG detector primarily arises from the calculation of the geometric center in (13). The computational complexities are $O(N_{R}n^{3})$ and $O(N_{R}n\log n)$, respectively. The number of merit functions in (22) that need to be calculated for preprocessing is $O(KN_{r}N_{a})$. After preprocessing *K* frames of data, the required computational complexity is $O(KN_{r}N_{a}N_{R}n^{3})$ and $O(KN_{r}N_{a}N_{R}n\log n)$.

During the integration of the merit function, the complexity for a single state transition is O(|τ|). The total of the $\left(K-1\right)N_{r}N_{a}$ merit function integration needs to be computed, so the complexity in the state transition stage is $O(\left(K-1\right)N_{r}N_{a}|\tau |)$. In the multi-target detection stage, the computational complexity for each integrated sub-region is $O(\left(K-1\right)m_{l}|\Omega_{l}||\tau |)$.

Therefore, the overall computational complexity of MIG-TBD is

(37) $$O\left(KN_{r}N_{a}N_{R}n^{3}\right)+O(\sum_{l=1}^{L}\left(K-1\right)m_{l}|\Omega_{l}\left||\tau |\right)$$

and the computational complexity of PSIG-TBD is

(38) $$O\left(KN_{r}N_{a}N_{R}n\log n\right)+O(\sum_{l=1}^{L}\left(K-1\right)m_{l}|\Omega_{l}||\tau |).$$

## 6. Experimental Results and Discussion

This section will validate the performance of the proposed method. The evaluation metrics used were as follows.

- The number of the valid targets $\hat{M}$: Estimated number of targets based on whether the threshold is exceeded [8].
- Detection probability $P_{d}$: The number of detected targets matches the actual number of targets, and the error between the position of the target in the last frame and the actual position is within two cells [4,8].
- Root mean square error of output trajectory position RMSE:
  (39) $$\mathrm{RMSE}=\sqrt{\frac{\sum_{m_{c}=1}^{M_{c}}\sum_{k=1}^{K}\left[{\left(r_{k}-{\hat{r}}_{k}\right)}^{2}+{\left(a_{k}-{\hat{a}}_{k}\right)}^{2}\right]}{M_{c}K}},$$
  where $M_{c}$ represents the number of Monte Carlo simulations.

The subsequent content will utilize sea clutter data to conduct experimental verification on typical parallel adjacent target and cross adjacent target scenarios. Subsequently, the detection and tracking results under complex multi-target scenarios are presented. For ease of comparison, the main comparison methods are based on square amplitude (SA) [48,49], the logarithm of the complex likelihood ratio (LCLR) [21], and fast Fourier transform constant false alarm rate (FFT-CFAR) [50]-based TBD algorithms.

### 6.1. Sea Clutter Data

**Figure 6:**
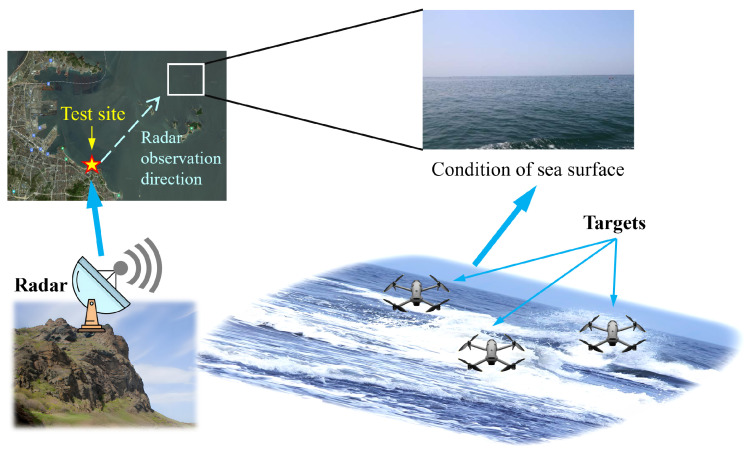
A scenario schematic diagram of the experiment.

The sea clutter data used in this paper was obtained from a sea detection experiment conducted using an X-band, solid-state, and fully phased array radar [51,52], as shown in Figure 6. This sea clutter data is intended to support research on sea clutter characteristics, the development of target detection algorithms, and radar performance optimization. The dataset includes sea clutter and target echo data under different sea conditions, resolutions, and ground elevation angles. The key radar parameters are shown in Table 1.

The simulated targets were injected into the recorded sea clutter data by adding coherent signal components, generated according to the target model described in (1), onto the clutter-dominated range-azimuth cells. The amplitude of each target followed a Gaussian distribution with a zero mean, and its variance was controlled to achieve the desired SCR. The target’s motion across frames followed the kinematic model outlined in (2) with specific parameter settings: the process noise intensity κ was randomly sampled from a uniform distribution over [−2.5, 2.5] m/s_2_, the initial velocity was uniformly distributed within [−30, 30] m/s, and the maximum permissible target velocity was constrained to 30 m/s. This configuration ensured realistic trajectory continuity and motion variability. The process allows for the simulation of fluctuating targets with glint-like amplitude behavior, reflecting the practical challenges of multi-target tracking in sea clutter. The SCR was set from 2 dB to 22 dB. Using the 10-frame echo measurements received by the radar, we extracted the observation region containing 50 range cells and 50 azimuth cells.

The false alarm rate is $P_{fa}=10^{-3}$. The reference cells were set to $N_{R}=8$, and each CPI contained n=8 pulses. The parameters of the algorithm proposed in this paper are ξ=0.1 and λ=5. Specifically, the used data files are 20191012110708_01_scanning.mat∼20191012110735_10_scanning.mat.

### 6.2. Performance and Efficiency Analysis Under Different Frame Counts

**Figure 7:**
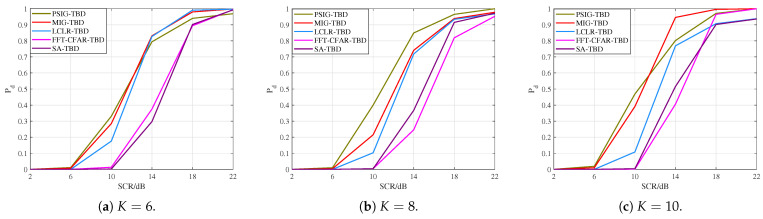
The detection performance curves with different numbers of frames.

To discuss the effect of the number of frames on the performance of the proposed algorithm, this subsection conducts experiments for K=6, K=8, and K=10 frames, respectively. Simulated targets, as described in Section 6.1, were added to the processed data, with two targets. After 1000 Monte Carlo simulations, the target detection probability curves were obtained, as shown in Figure 7. By comparing the detection performance, it can be found that the detection performance of PSIG-TBD and MIG-TBD continued to improve as the number of frames increased. Furthermore, compared with the reference methods, the performance improvement brought by the increased number of frames was more significant for the proposed methods. This also demonstrated the promoting effect of increasing frame numbers on detection performance. Moreover, as the number of frames increased, the performance improvement of MIG-TBD became even more significant.

Simultaneously, we analyzed the computational complexity of the five methods during single-frame processing, as shown in Table 2. In the proposed methods, the complexity of MIG-TBD is relatively high due to matrix operations. This means that increasing the number of frames will lead to continuously rising computational complexity for the proposed algorithm. The complexity primarily stems from the covariance matrix operations in MIG-TBD ($O(n^{3})$) and the geometric mean calculations. However, the PSIG-TBD variant offers reduced complexity (O(nlogn)) through power spectrum transformation. This performance–complexity trade-off is justified by the algorithm’s ability to address challenging multi-target tracking conditions that conventional methods cannot effectively handle. Increasing the number of frames provides limited performance improvement. The performance gain diminishes as more frames are added. Therefore, in comprehensively balancing the impact of detection performance and computational complexity, subsequent experiments will select 10 frames as the number of frames for multi-frame processing.

### 6.3. Parallel Adjacent Targets

**Figure 8:**
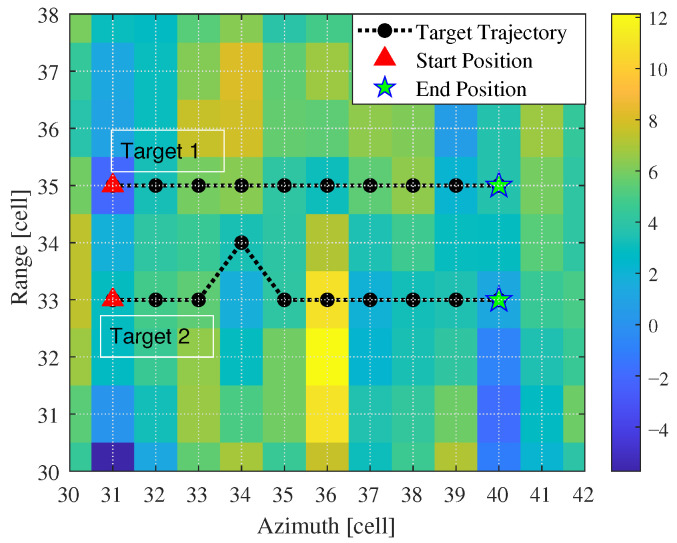
Parallel adjacent target motion scene.

**Figure 9:**
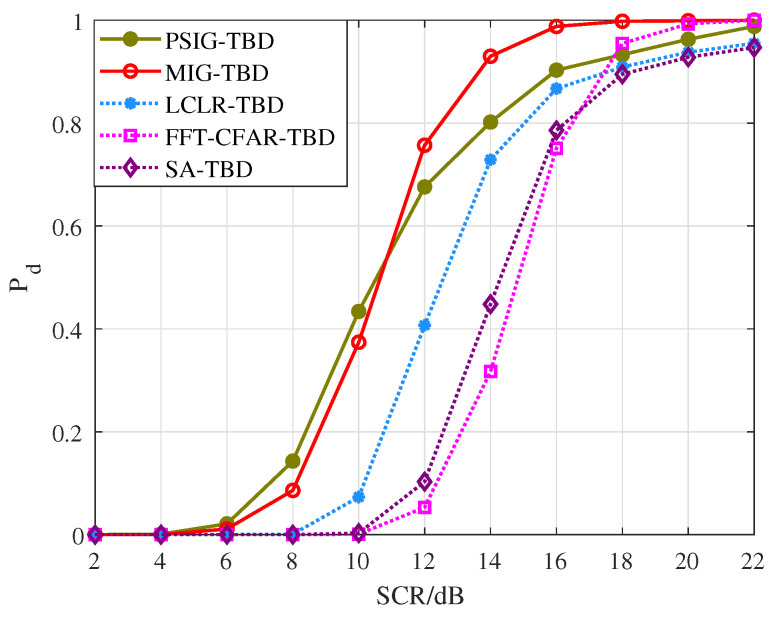
$P_{d}$ versus SCR for parallel targets.

**Figure 10:**
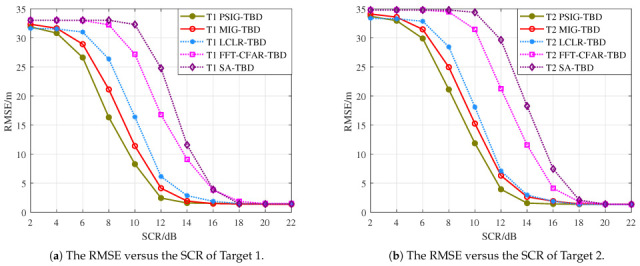
RMSE curves for each target in the parallel adjacent target motion scene.

**Figure 11:**
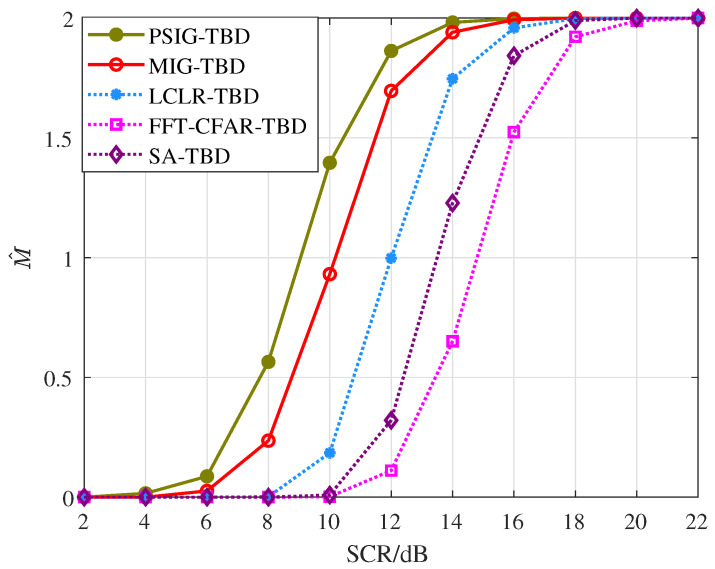
Number of valid targets declared.

First, we validated the detection and tracking performance of the proposed method for scenarios involving parallel adjacent targets. Simulated targets were added to each frame, totaling 10 frames. The distance between targets was within two range cells, as shown in Figure 8. During processing, the number and positions of targets were unknown. After 1000 Monte Carlo simulations, we obtained the detection probability curves and RMSE curves for each target, as shown in Figure 9 and Figure 10, where T1 and T2 represent Target 1 and Target 2, respectively. Additionally, we counted the number of valid targets declared, as shown in Figure 11.

From the perspective of detection probability $P_{d}$, the proposed methods PSIG-TBD and MIG-TBD demonstrate superior detection performance. Overall, MIG-TBD exhibits more robust detection performance for parallel adjacent targets. As shown in Figure 9, when SCR = 14 dB, the detection probability of MIG-TBD has already exceeded 90%. At this point, the detection probability of PSIG-TBD is 80%. When comparing the three comparison methods, LCLR-TBD, FFT-CFAR-TBD, and SA-TBD, the best-performing method is LCLR-TBD. At a detection probability of 90%, MIG-TBD achieves an SCR improvement of approximately 3 dB compared to LCLR-TBD. Compared to FFT-CFAR-TBD and SA-TBD, the SCR improvements are approximately 4 dB and 5 dB, respectively. From the tracking accuracy in Figure 10, PSIG-TBD and MIG-TBD have a smaller RMSE compared to the comparison methods. This indicates that the proposed methods also have higher tracking accuracy compared to traditional methods. Among these, PSIG-TBD has higher tracking accuracy. Benefiting from the superior discriminative capability of IG detectors for adjacent targets, both PSIG-TBD and MIG-TBD algorithms generate target trajectories with fewer false alarms, consequently achieving smaller RMSE values.

Figure 11 demonstrates the target number estimation performance of the proposed methods. As the SCR increases, the number of targets declared by different methods gradually grows. Among these, PSIG-TBD demonstrates superior estimation performance. When the SCR reaches 14 dB, the PSIG-TBD method can reliably detect two adjacent targets, with MIG-TBD showing comparable capability. To achieve the same detection performance, LCLR-TBD, FFT-CFAR-TBD, and SA-TBD require SCR levels of 16 dB, 18 dB, and 20 dB, respectively.

### 6.4. Cross Adjacent Targets

**Figure 12:**
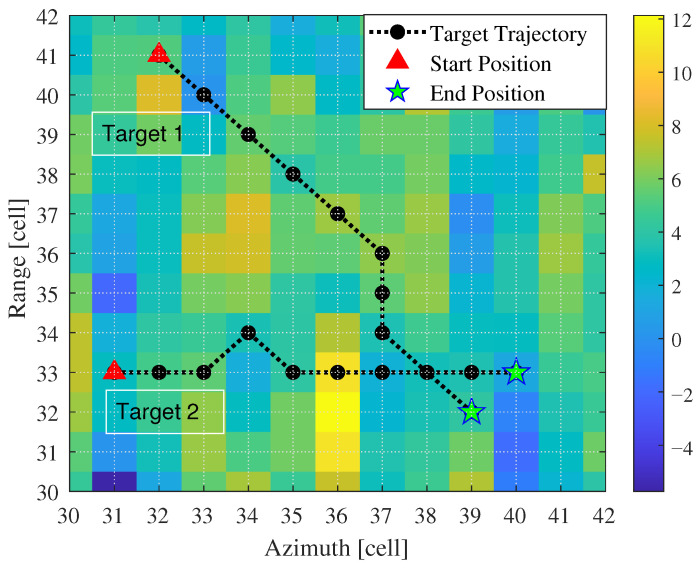
Cross adjacent target motion scene.

For multi-target detection, cross adjacent targets pose a significant challenge, as trajectory crossings may lead to tracking mismatches and degrade tracking accuracy. This section establishes a target motion scenario with cross adjacent targets, as illustrated in Figure 12. Target 1 and Target 2 pass through the same resolution cell at the 9th frame and 8th frame, and they remain in adjacent states during the final five frames. Simulation experiments were conducted to statistically analyze the detection and tracking curves of the different methods, along with target number estimation.

**Figure 13:**
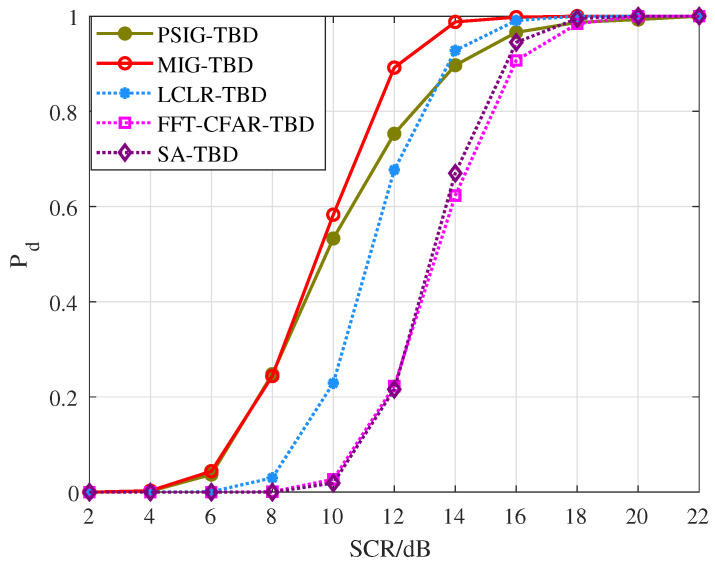
$P_{d}$ versus SCR for cross targets.

**Figure 14:**
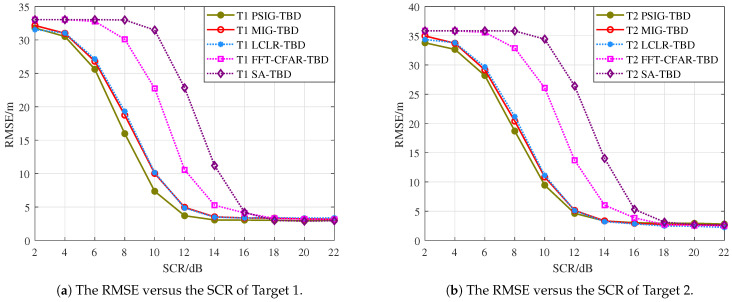
RMSE curves for each target in the cross adjacent target motion scene.

Figure 13 presents the target detection probability curves in crossing-parallel scenarios. The results demonstrate that both proposed methods, PSIG-TBD and MIG-TBD, exhibit superior detection performance compared to comparison methods. Notably, MIG-TBD shows a particularly outstanding performance. At SCR = 12 dB, MIG-TBD achieves a 90% detection probability, representing improvements of 2 dB, 4 dB, and 4 dB over LCLR-TBD, FFT-CFAR-TBD, and SA-TBD, respectively. Figure 14 also compares the tracking accuracy in terms of RMSE. PSIG-TBD, MIG-TBD, and LCLR-TBD show significantly lower RMSE values than FFT-CFAR-TBD and SA-TBD, with PSIG-TBD demonstrating the best performance, followed by MIG-TBD. A cross-comparison revealed that the convergence values of the RMSE curves varied across different target scenarios. As shown in Figure 10, the RMSE converges at approximately 1.4 m, while, in Figure 14, it converges around 2.6 m. This discrepancy arises from the differences in estimation errors for distinct target trajectories.

**Figure 15:**
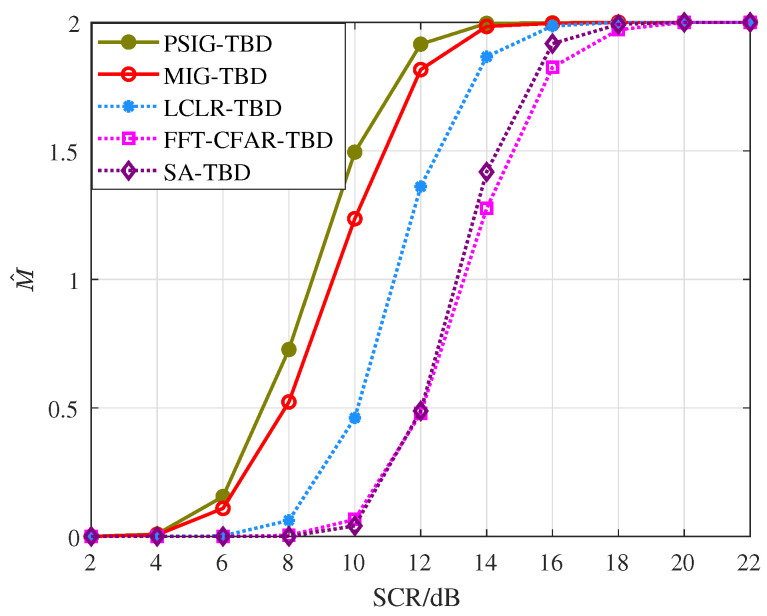
Number of valid targets declared.

Figure 15 illustrates the target number estimation performance in the crossing parallel scenarios. The proposed methods maintain excellent resolution capability, with both PSIG-TBD and MIG-TBD successfully detecting two targets at SCR = 14 dB. In contrast, LCLR-TBD, FFT-CFAR-TBD, and SA-TBD failed to accurately declare all the valid targets at low SCR due to interference from adjacent targets. These results confirm that the proposed methods effectively enhance both detection and tracking performance.

### 6.5. Complicated Multi-Target Scenario

**Figure 16:**
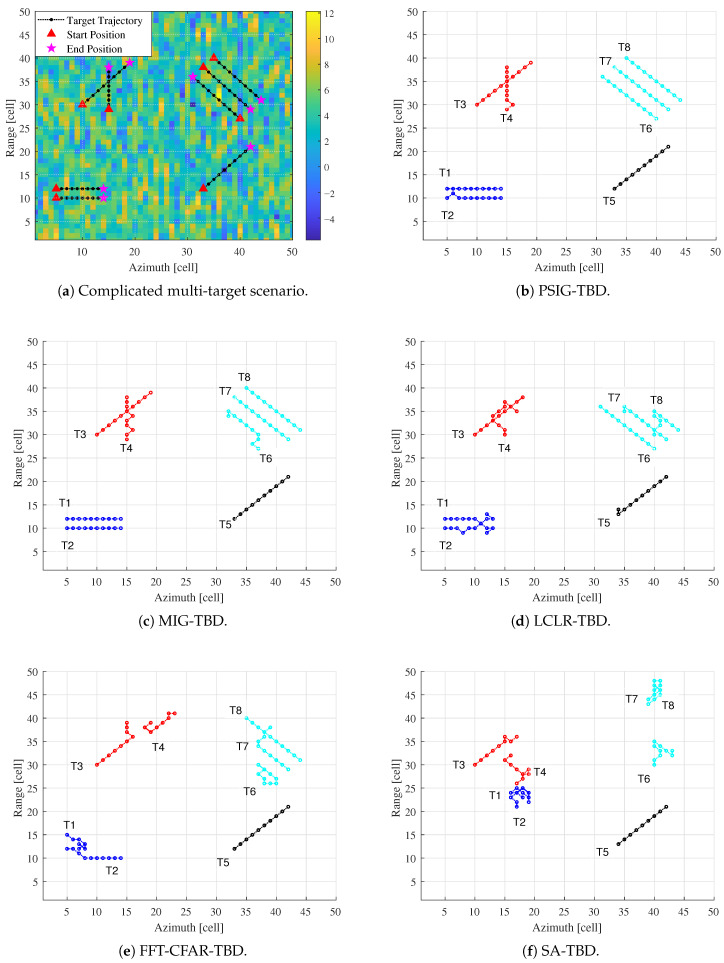
Tracking results of the proposed method versus comparative methods in complex multi-target scenarios.

The proposed multi-target TBD algorithm’s detection and tracking performance in complex multi-target scenarios was verified by considering a scenario containing eight moving targets in this subsection. The scenario is shown in Figure 16a, where triangles represent the targets’ positions in the first frame and pentagrams represent their positions in the 10th frame. The SCR was set to 12 dB.

In this scenario, Target 1 (T1) and Target 2 (T2) are parallel adjacent targets separated by two resolution cells. Target 3 (T3) and Target 4 (T4) are crossing adjacent targets that share the same resolution cell in the sixth frame and seventh frame. Target 5 (T5) is an isolated target. Target 6 (T6), Target 7 (T7), and Target 8 (T8) are three parallel adjacent targets separated by two resolution cells. Following the proposed multi-target TBD processing flow, adjacent targets can be clustered and partitioned into sub-regions. The processing results of the proposed method and the comparison methods are shown in Figure 16.

The proposed PSIG-TBD and MIG-TBD methods demonstrate robust detection capabilities, successfully detecting all eight targets with high trajectory accuracy. The primary tracking errors occurred for Targets T2, T4, and T6. These inaccuracies arise mainly during target crossing events and in regions with parallel, closely-spaced trajectories, where data association becomes ambiguous due to overlapping kinematic signatures. Despite these challenging scenarios, both PSIG-TBD and MIG-TBD output trajectories with acceptable deviations, underscoring their overall superior performance. In contrast, LCLR-TBD exhibits significant deviations in tracking T4 and T8 compared to the truth trajectories, with T1 and T2 showing trajectory crossover. FFT-CFAR-TBD only maintains satisfactory trajectory integrity for T5 and T8, while other targets show substantial tracking errors. SA-TBD performs even worse, with only T3 and T5 being tracked completely. The primary reason stems from strong clutter interference, which causes the trackers to lock onto high-clutter regions, resulting in false trajectories.

## 7. Conclusions

This paper investigated the problem of detecting and tracking complex multi-target in sea clutter environments. Based on IG theory and multi-frame TBD techniques, we designed a multi-frame, multi-target detector using information geometry. Specifically, to address the challenge of effectively distinguishing adjacent multiple targets, we developed a joint merit function incorporating both transition cost and feature dissimilarity, building upon the fundamental principles of IG detectors. The proposed approach processes the multi-target problem through DP and the greedy integration algorithm for recursive multi-frame measurement integration, followed by target cancellation procedures. Experimental results using real sea clutter data demonstrate that the proposed PSIG-TBD and MIG-TBD methods achieve superior multi-target detection performance and higher tracking accuracy for adjacent targets compared to conventional approaches, such as LCLR-TBD. The SCR is improved by at least 2 dB.

Despite promising results, this study has limitations that suggest future research directions. The computational complexity of MIG-TBD remains challenging for real-time applications, requiring more efficient approximation algorithms. Additionally, theoretical analysis can be conducted on the statistical properties of the information geometry-based merit function to facilitate the application of the proposed algorithm.

## Figures and Tables

**Table 1:** Radar key parameters [52].

Parameters	Values
Operating Frequency Band	X-band (9.3∼9.5 GHz)
Transmission Scheme	Single carrier + LFM signal
Pulse Repetition Frequency	1.6 kHz, 3 kHz, 5 kHz, 10 kHz
Range Resolution	6 m (LFM pulse)
Peak Power	50 W
Horizontal Beamwidth	$1.2^{\circ }$
Vertical Beamwidth	$22^{\circ }$
Polarization Mode	HH polarization
Sampling Rate	60 MHz (LFM signal)
Data Quantization	14-bit ADC

**Table 2:** Computational complexity comparison.

Method	Complexity per Frame
SA-TBD	$O(N_{r}N_{a})$
FFT-CFAR-TBD	$O(N_{r}N_{a}\log n)$
LCLR-TBD	$O(N_{r}N_{a}n^{2})$
MIG-TBD (proposed)	$O(N_{r}N_{a}N_{R}n^{3})$
PSIG-TBD (proposed)	$O(N_{r}N_{a}N_{R}n\log n)$

## Data Availability

The datasets analyzed during this study are available at https://radars.ac.cn/web/data/getData?dataType=DatasetofRadarDetectingSea (accessed on 15 June 2025).
